# Antibacterial Activity of a Fractionated *Pistacia lentiscus* Oil Against Pharyngeal and Ear Pathogens, Alone or in Combination With Antibiotics

**DOI:** 10.3389/fmicb.2021.686942

**Published:** 2021-06-17

**Authors:** Francesco Di Pierro, Valeria Sagheddu, Serena Galletti, Mara Forti, Marina Elli, Alexander Bertuccioli, Simone Gaeta

**Affiliations:** ^1^Velleja Research, Milan, Italy; ^2^Digestive Endoscopy Unit and Gastroenterology, Fondazione Poliambulanza, Brescia, Italy; ^3^AAT-Advanced Analytical Technologies, Piacenza, Italy; ^4^Department of Biomolecular Sciences (DISB), University of Urbino, Urbino, Italy; ^5^Dipartimento di Scienze del Farmaco, Università del Piemonte Orientale, Novara, Italy

**Keywords:** winterizing, lentisk oil, *S*. *pyogenes*, *S*. *pneumoniae*, *M*. *catarrhalis*, *H*. *influenzae*, *S*. *salivarius* K12, *S. salivarius* M18

## Abstract

Previous studies have clearly demonstrated that the addition of lentisk oil (LO) to streptococcal cultures makes it possible to differentiate *Streptococcus* spp. into three categories with *Streptococcus mitis* and *Streptococcus intermedius* sensitive, *Streptococcus pyogenes*, *Streptococcus agalactiae*, and *Streptococcus mutans* partially sensitive, and *Streptococcus salivarius* insensitive to the product. We have investigated here whether the winterization of LO, an easy and cheap procedure that removes some of the fatty substances contained within, resulted in a better antimicrobial effect on human pathogens affecting the pharyngeal mucosa and middle ear such as *S*. *pyogenes*, *S*. *pneumoniae*, *Moraxella catarrhalis*, and *Haemophilus influenzae*, without affecting, or minimally affecting, *S*. *salivarius* strains, oral probiotics commonly used to reduce oral and middle ear infection recurrence, especially in children. Our results not only demonstrated a stronger antimicrobial action of winterized LO (WLO) on *S*. *pyogenes*, compared to what was seen with LO, but also demonstrated a strong antimicrobial action vs. *S*. *pneumoniae* and *M*. *catarrhalis* and a very limited effect on *S*. *salivarius* (strains K12 and M18). Moreover, WLO demonstrated a co-acting action when tested along with the antibiotics amoxicillin (A) and amoxicillin clavulanate (AC), effects clearly visible also on *H*. *influenzae*. Our results also showed that at least part of the antimicrobial effect observed was due to the presence of anacardic acids (AAs). Finally, WLO, when tested with human peripheral blood mononuclear cells (h-PBMCs), reduced the release of IL-6 and TNF-α and, in the case of cells stimulated by LPS, the release of IFN-γ. In conclusion, our study highlights an enhanced antimicrobial role for LO when winterized, suggests a co-acting effect of this when given with antibiotics, identifies AAs as possible active ingredients, and proposes a possible anti-inflammatory role for it.

## Introduction

In Western countries, acute otitis media (AOM) and pharyngo-tonsillitis (Ph-T) are among the most common reasons for ambulatory visits and antibiotic prescription in pediatric primary care (Hersh et al., 2011). Several risk factors promote AOM and Ph-T, including prematurity, preschool age, early attendance at nursery school, air pollution, home dampness, passive exposure to tobacco, or vape fumes, a low socio-economic level, overcrowding, and allergy (Patria and Esposito, 2013). Although viral infections very often represent an important, or the initial, cause of pathology, bacterial superinfections may be often associated, entailing the frequent use of antibiotics (Li et al., 2016). Even though it is not generalizable to all bacteria and antibiotics, antibiotic resistance could be considered a significant problem (Guitor and Wright, 2018). Bacteria are also known to create protecting biofilms, resulting in lack of success for antibiotics and pathogen survival by also promoting unfavorable host–bacteria interactions (Drago et al., 2019). The bacteria more often involved in these diseases, particularly in cases of recurrency, are *Streptococcus pyogenes*, mainly in cases of Ph-T infection, and *Haemophilus influenzae*, *S*. *pneumoniae*, and *Moraxella catarrhalis*, mainly in cases of AOM (Klein, 1997). Recently, oral probiotics have been tested to prevent recurrent episodes of AOM and/or Ph-T, to decrease the use of antibiotic therapy, to reduce the possible incidence of drug resistance, as well as to reduce the need for tonsillectomy and adenoidectomy (Marini et al., 2019). The success observed can be explained by considering the role played by “health-friendly bacteria,” such as *Streptococcus salivarius* K12, in releasing lantibiotics, these last being able to counteract and halt the presence of oral and ear pathogens (Wescombe et al., 2012). Recently, in an attempt to highlight new therapeutic strategies for novel antimicrobials, a “lentisk oil–bacteria” interaction has been hypothesized, with particular emphasis on different *Streptococcus* species, some of which at least are also involved in pharyngeal diseases (Orrù et al., 2017). Lentisk (*Pistacia lentiscus*) oil (LO) represents a typical vegetal product of the Mediterranean basin that has been used in traditional cuisine for hundreds of years, at least within some Mediterranean countries (Italy, Greece, and Tunisia, among others). Besides its nutritional properties, LO could represent an interesting candidate for use as an anti-infective agent. In fact, it is also widely used in Mediterranean folk medicine for some affections such as acute gingivitis, pediatric skin infections, mainly impetigo and foot plaques, and biofilm-related infections often associated with *Streptococcus* spp. (Siano et al., 2020). Some researchers have observed that the addition of LO to streptococcal cultures allows differentiation of the *Streptococcus* genus into LO-sensitive (*Streptococcus mitis* and *Streptococcus intermedius*), LO-partially sensitive (*S*. *pyogenes*, *Streptococcus agalactiae*, and *Streptococcus mutans*), and LO-insensitive (*S*. *salivarius*) species (Orrù et al., 2017). The result was correlated by the authors with the fatty acid metabolic pathways of the species considered. In fact, analysis of the growth medium where *Streptococcus* spp. were cultured along with LO detected a significant increase in the free unsaturated fatty acids (UFAs), oleic and linoleic acid, which indeed had already been described for their antibacterial activity (Speert and Wannamaker, 1980). *P*. *lentiscus* belongs to Anacardiaceae, a botanical family known to contain anacardic acids (AAs), mainly AA, cardanol, and cardol, endowed with recognized antibiotic activity (Hamad and Mubofu, 2015). Moreover, derivatives and analogs of AAs are also endowed with antibacterial actions (Mamidyala et al., 2013), and recently, AA impregnation of catheter surfaces for the prevention of *Staphylococcus aureus* attachment and biofilm formation was shown to be successful (Sajeevan et al., 2018). Similarly, LO, that is the oil produced by pressing lentisk berries at room temperature, has been reported to have antimicrobial activity against *S*. *aureus* (Mezni et al., 2015). Based on the above evidence, without denying the possible antibiotic role played by UFAs as described by Orrù et al. (2017), we considered that at least part of the anti-streptococcal effect observed for LO was due to the presence of AAs. The primary aim of this study was therefore to investigate the ability of both winterized LO (WLO) and AAs purified from this to negatively affect human streptococcal pathogens, such as *S*. *pyogenes*, and human ear pathogens, such as *S*. *pneumoniae*, *M*. *catarrhalis*, and *H*. *influenzae*, when used alone or combined with amoxicillin (A) or amoxicillin clavulanate (AC). The secondary aim of our study was to assess, for both WLO and AAs, the impact on cytokine release by a cultured human cell line, in basal conditions and following the induction of an inflammatory state by LPS.

## Materials and Methods

### WLO

Lentisk oil was obtained from *P*. *lentiscus* fruit from Mediflora^®^ (Pula, Cagliari, Italy) by using ripe berries harvested in winter in the southern Sardinian region (specific weight = 890 kg/m^3^). According to the LO manufacturer, after a cycle of light dehydration by air for 20 days, the drupes were cold pressed following an already described method (Mezni et al., 2014). The LO was then filtered and stored in stainless steel containers at 20°C until bottling. Prior to testing, the sample was centrifuged at 12,000 rpm for 15 min and the supernatant was subjected to winterization, according to an already described method (Nergiz and Celikkale, 2011), to remove fatty substances and impurities and further stabilize its condition (Watari et al., 2020).

### Extraction of AAs From WLO

**FIGURE 1:**
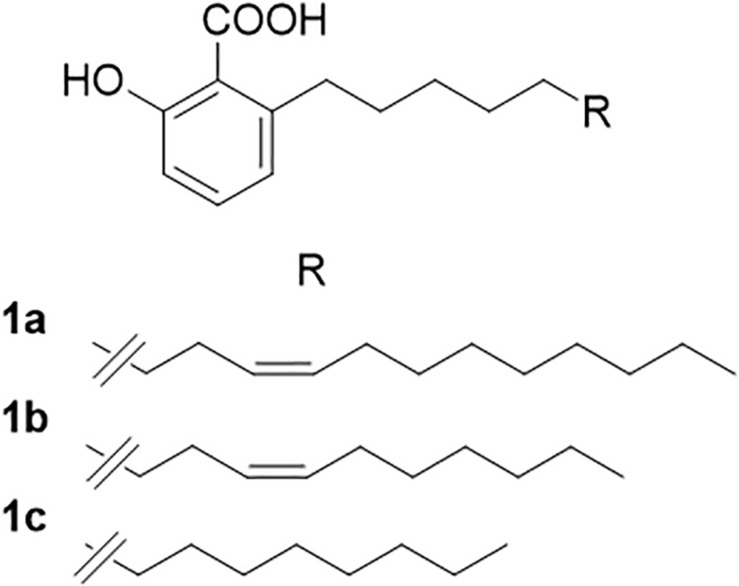
Anacardic acids from lentisk (*Pistacia lentiscus* L.) oil. **(A)** C17:1; anacardic acid; 49% of the mixture. **(B)** C15:1; ginkgolic acid-1; 36% of the mixture. **(C)** C13:0; anacardic acid; 9% of the mixture.

A sample (100 mL) of the WLO was dissolved in methanol and cooled to −18°C overnight. The copious precipitate of triglycerides was filtered off under vacuum. The precipitate was washed with cold (−18°C) methanol, and the pooled filtrates were evaporated to afford 4.1 g (4.1%) of a thick oily residue. Purification by gravity column chromatography was unsuccessful under silica gel and RP18 conditions, and therefore, a sample of the WLO (2 g) was transesterified with HCl in methanol (1 N, from SO_2_Cl_2_ and dry methanol). After stirring at room temperature for 2 h, the reaction was worked-up by neutralization with the careful addition of solid NaHCO_3_. After filtration and evaporation, the residue was purified by gravity column chromatography on silica gel (50 g), using 5% acetic acid in petroleum ether–ethyl acetate (98:2) as an eluant to afford a mixture of AAs (220 mg, 0.45%) as a semi-solid white paste. Analysis by HPLC-MS (Morais et al., 2017) showed a mixture of three AAs (Figure 1). The major AA (C17:1; AA) accounted for 49% of the mixture. The product also contained significant amounts of its smaller analog (C15:1; ginkgolic acid-1; 36% of the mixture), with minor amounts of the fully saturated analog (C13:0; AA; 9% of the mixture).

### Bacterial Strains and Culture Conditions

The strains included within the study were: *S*. *salivarius* K12 (ATCC BAA-1 024), *S*. *salivarius* M18 (DSM 14865), both isolated from commercially available probiotics, respectively, named Bactoblis^®^ and Carioblis^®^ (Pharmextracta, Italy), and *S*. *pyogenes* LMG 21599, *M*. *catarrhalis* DSM 9143, *S*. *pneumoniae* DSM 20566, and *H*. *influenzae* DSM 4690 obtained from the International Depository Authorities (IDAs), the Belgian Co-ordinated Collections of Micro-organisms (BCCM/LMG), and the German Collection of Microorganisms and Cell Cultures (DSMZ).

Both *S*. *salivarius* K12 and M18 strains, *S*. *pyogenes* LMG 21599, *M*. *catarrhalis* DSM 9143, and *S*. *pneumoniae* DSM 20566 were subcultured on BHI agar while *H*. *influenzae* DSM 4690 was subcultured on chocolate agar. Plates were incubated for 48 h at 37°C under microaerophilic conditions or under anaerobic atmosphere for *H*. *influenzae* DSM 4690.

### Growth Inhibition Assay

The minimal inhibitory concentration (MIC) was assessed by the macrodilution method, in accordance with CLSI M07 “Methods for Dilution Antimicrobial Susceptibility Tests for Bacteria That Grow Aerobically”; Approved Standard—9th Edition, with minor modifications. Following the method previously reported by Orrù et al. (2017), macrodilutions were carried out by evaluating decreasing concentrations of test substances mixed with BHI broth (Oxoid, Rodano, Italy). *H*. *influenzae* DSM 4690 was tested using BHI with the addition of 0.1% sterile defibrinated horse blood (BHI-mod). Substances to be tested were added, alone or in combination, to BHI broth as reported in Table 1. The final concentrations (vol/vol) of the substances tested in combination, such as the antibiotics plus WLO and the antibiotics plus AAs, were selected based on the sensitivity results obtained by testing pure substances. BHI and BHI-mod without any additives were used as negative controls. Amoxicillin (A) and AC (both from Sigma-Aldrich, United States) were freshly prepared at a stock concentration of 1 mg/mL and sterilized by filtration; WLO was directly used without any further manipulation; AAs were maintained at 4°C until use and firstly solubilized in dimethyl sulfoxide (DMSO) and then in sterile water. Colonies from agar plates were isolated, resuspended in saline solution, and optically adjusted by the 0.5 McFarland standard corresponding to 1.5 × 10^8^ CFU/mL, and diluted 10-fold to a concentration of 10^7^ CFU/mL. A 100-fold dilution of each suspension was then inoculated in the test tubes. All tubes were incubated for 48 h at 37°C under microaerophilic conditions or under anaerobic atmosphere for *H*. *influenzae* DSM 4690. MIC values were recorded as the lowest concentration without visible microorganism growth under a white light microscope.

**TABLE 1:** Tested concentrations.

**Strain**	**Added substance**	**Final concentrations (μg/mL)**
	WLO	400–125,000
*S*. *salivarius* K12	A	0.02–1.28
*S*. *salivarius* M18	AC	0.03–2
*S*. *pyogenes* LMG 21599	AAs	0.03–512
*M*. *catarrhalis* DSM 9143	A + WLO	0.02 + 3,900; 0.04 + 7,800; 0.08 + 15,600
*S*. *pneumoniae* DSM 20566	AC + WLO	0.015 + 800; 0.03 + 1,900; 0.06 + 3,800
	A + AAs	0.02 + 0.25; 0.04 + 0.5; 0.08 + 1
	AC + AAs	0.015 + 0.25; 0.03 + 0.5; 0.06 + 1
	WLO	400–125,000
	A	0.02–1.28
	AC	0.03–2
	AAs	0.03–512
	A + WLO	0.32 + 31,200; 0.64 + 31,200; 1.28 + 31,200
*H*. *influenzae* DSM 4690		0.32 + 62,500; 0.64 + 62,500; 1.28 + 62,500
	AC + WLO	0.25 + 31,200; 0.5 + 31,200; 1 + 31,200
		0.25 + 62,500; 0.5 + 62,500; 1 + 62,500
	A + AAs	0.32 + 256; 0.64 + 256; 1.28 + 256
		0.32 + 512; 0.64 + 512; 1.28 + 512
	AC + AAs	0.25 + 256; 0.5 + 256; 1 + 256
		0.25 + 512; 0.5 + 512; 1 + 512

*WLO, winterized lentisk oil; A, amoxicillin; AC, amoxicillin clavulanate; AAs, anacardic acids.*

### Evaluation of the Anti-Inflammatory Properties of WLO and AAs

According to the manufacturer’s (Lonza, Italy; Catalog N°CC-2702) instructions, human peripheral blood mononuclear cells (h-PBMCs) were immediately plated in a 24-well plate at a concentration of 1 × 10^6^ cells/mL in RPMI 1640 medium (Euroclone, Italy) supplemented with 10% heat-inactivated FBS (fetal bovine serum, Euroclone), 2 mM L-glutamine, and 50 μg/mL gentamicin. The evaluation of the immune-modulatory potential of WLO and AAs was conducted under basal conditions, in order to exclude potential intrinsic inflammatory features, and in the presence of LPS at 100 ng/mL. After 24 h of co-incubation, supernatants were collected and used for cytokine quantification. IL-6, TNF-α, and IFN-γ were quantified using specific ELISA kits (Thermo Fisher Scientific, MA, United States). In addition to the control (RPMI only plus FBS) we tested WLO from 10 to 40 μg/mL and AAs from 0.015 to 0.060 μg/mL, with or without LPS. Each condition was tested in triplicate. To evaluate the viability of the cells the trypan blue exclusion assay was performed for each tested condition.

### Statistical Analysis

Statistical analysis included a one-way ANOVA (analysis of variance) and at *p* < 0.05 Dunnett’s *post hoc* test was performed. All statistics were carried out using Prism version 8.

## Results

**FIGURE 2:**
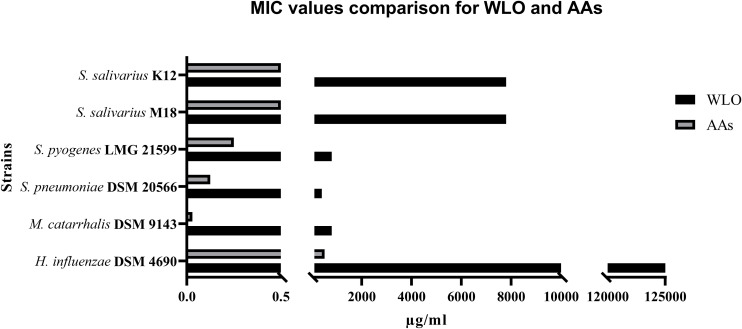
Comparison between MIC values obtained for winterized lentisk oil (WLO) and anacardic acids (AAs) (μg/mL).

The main aim of our study was to confirm the antibacterial potential of LO, as already demonstrated for some human streptococcal pathogens (Orrù et al., 2017), and to investigate the possibility of enhancing this by exploiting its main components and testing along with antibiotics such as A and AC. Assuming that the actions of LO could be due to the presence of AAs, we increased the concentration of AAs in the oil by a process called winterization, known to be a simple and cheap method of reducing the fatty acid and triglyceride content of oils. The concentration ranges tested for any single ingredient vs. any single strain are shown in Table 1. The antibiotic activity of WLO, A, AC, and AAs was assessed on strains of *S*. *pyogenes* LMG 21599, *S*. *pneumoniae* DSM 20566, *M*. *catarrhalis* DSM 9143, and *H*. *influenzae* DSM 4690, bacterial species known to be involved in recurrent pharyngeal and middle ear pathologies especially affecting children. We also tested the effects of these actives on oral probiotics (*S*. *salivarius*, strains K12 and M18) previously proven to be competitive with the above listed pathogens (Wescombe et al., 2012). The MIC values obtained are reported in Table 2. The two probiotics, *S*. *salivarius* K12 and *S*. *salivarius* M18, and the pathogen *H*. *influenzae* DSM 4690, showed the highest tolerance to WLO, with values of 0.78% for both probiotics and 12.5% for DSM 4690. Lower MICs were observed for *S*. *pyogenes* LMG 21599, *S*. *pneumoniae* DSM 20566, and *M*. *catarrhalis* DSM 9143 (between 0.04 and 0.08%). Similarly, the greatest MIC values were observed for *H*. *influenzae* DSM 4690 vs. A, AC, and AAs. This result appears to be in line with published data that reported for *H*. *influenzae* a well-recognized β-lactamase action against A (García-Cobos et al., 2008). As expected, no relevant differences in terms of the MIC were observed for the other tested strains when treated with A and AC, although very slightly higher MICs were observed for the probiotics K12 and M18. Interestingly, the data presented in Table 2 show that the MIC values obtained for AAs were significantly lower than those found for WLO, and that the pathogens were found to be more sensitive to AAs than to WLO. According to these data, it seems that an AA mixture from the lentisk berry has antimicrobial activity toward pathogens. Probiotic *S*. *salivarius* K12 and M18 showed a better tolerance to AAs than *M*. *catarrhalis* DSM 9143 and pathogenic streptococci. *H*. *influenzae* DSM 4690 seemed to be insensitive to AAs and to elevated concentrations of WLO. A graphical representation of the comparison between results obtained for WLO and AAs is given in Figure 2.

**TABLE 2:** MIC values obtained for all tested strains.

**Strain**	**A (μg/mL)**	**AC (μg/mL)**	**WLO (μg/mL)**	**AAs (μg/mL)**
*S*. *salivarius* K12	0.08	0.06	7,800	0.5
*S*. *salivarius* M18	0.08	0.06	7,800	0.5
*S*. *pyogenes* LMG 21599	0.08	0.03	800	0.25
*S*. *pneumoniae* DSM 20566	0.04	0.03	400	0.125
*M*. *catarrhalis* DSM 9143	0.02	0.03	800	0.03
*H*. *influenzae* DSM 4690	1.28	2.0	125,000	512

*MIC, minimal inhibitory concentration; WLO, winterized lentisk oil; A, amoxicillin; AC, amoxicillin clavulanate; AAs, anacardic acids.*

Despite the interesting ability of WLO and its component AAs to negatively affect the growth of most of the tested oral pathogens, the results were not obviously comparable to the inhibitory effects of the antibiotics A and AC. Therefore, we investigated the possibility of constraining the use of antibiotics by exploiting their potential synergy with WLO and/or AAs. The effects of WLO or AAs plus A or AC are shown in Table 3. Based on these results, it was possible to confirm the synergistic effects of the two substances when used together with antibiotics against oral and ear pathogens. For WLO, this was more noticeable when AC was added than when A was added since the MIC values for all pathogenic strains, except for *H*. *influenzae*, were reached with a lower concentration of WLO when combined with AC compared to A. Regarding the AAs, we did not observe clear differences between A and AC when they were added to AA, and all strains, including *H*. *influenzae*, showed similar results.

**TABLE 3:** MIC values obtained for the combination of antibiotics with winterized lentisk oil and antibiotics with anacardic acids.

**Strain**	**A (μg/mL) + WLO (μg/mL)**	**AC (μg/mL) + WLO (μg/mL)**	**A (μg/mL) + AAs (μg/mL)**	**AC (μg/mL) + AAs (μg/mL)**
*S*. *salivarius* K12	0.04 + 7,800	0.03 + 1,900	0.04 + 0.25	0.03 + 0.25
*S*. *salivarius* M18	0.04 + 7,800	0.03 + 1,900	0.04 + 0.5	0.03 + 0.5
*S*. *pyogenes* LMG 21599	0.02 + 3,900	0.015 + 800	0.02 + 0.125	0.015 + 0.125
*S*. *pneumoniae* DSM 20566	0.02 + 3,900	0.015 + 800	0.02 + 0.125	0.015 + 0.125
*M*. *catarrhalis* DSM 9143	0.02 + 3,900	0.015 + 800	0.02 + 0.06	0.015 + 0.06
*H*. *influenzae* DSM 4690	1.28 + 31,200	1.0 + 62,500	0.64 + 256	0.5 + 256

*MIC, minimal inhibitory concentration; WLO, winterized lentisk oil; A, amoxicillin; AC, amoxicillin clavulanate; AAs, anacardic acids.*

To assume if the role played by WLO and/or AAs on the tested strains were bactericidal or bacteriostatic, we have also tested in our *in vitro* model the MBC (minimal bactericidal concentration) values (Table 4). As it can be observed by a direct comparison with the MIC values reported in Table 2, for the two probiotic strains (K12 and M18) the MBC is higher than the MIC indicating that the WLO and the AAs act against them mainly as bacteriostatic agents. Differently, with regards to the pathogen strains, the actives seem to act mainly as bactericidal as the MIC coincides with the MBC. This does not apply to *H. influenzae* which is poorly sensitive and out of the range with respect to the other microorganisms analyzed.

**TABLE 4:** MBC values obtained for all tested strains.

**Strain**	**A (μg/mL)**	**AC (μg/mL)**	**WLO (μg/mL)**	**AAs (μg/mL)**
*S*. *salivarius* K12	0.08	0.06	15,600	1
*S*. *salivarius* M18	0.04	0.03	15,600	1
*S*. *pyogenes* LMG 21599	0.04	0.03	800	0.25
*S*. *pneumoniae* DSM 20566	0.04	0.03	400	0.125
*M*. *catarrhalis* DSM 9143	0.02	0.03	800	0.03
*H*. *influenzae* DSM 4690	1.28	2	125,000	512

*MBC, minimal bactericidal concentration; WLO, winterized lentisk oil; A, amoxicillin; AC, amoxicillin clavulanate; AAs, anacardic acids.*

**FIGURE 3:**
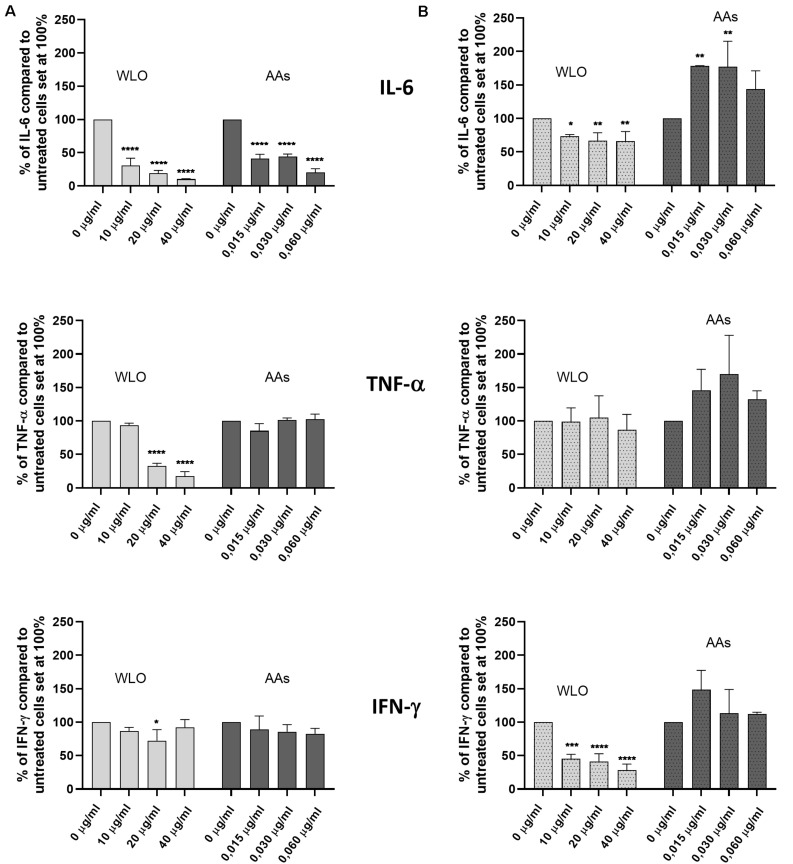
Effects of winterized lentisk oil (WLO) and anacardic acids (AAs) on IL-6, TNF-α, and IFN-γ release from human peripheral blood mononuclear cells (h-PBMCs) in both **(A)** basal and **(B)** LPS-stimulated conditions. Statistical analysis was performed using one-way ANOVA (vs. untreated cells) (*p < 0.05, **p < 0.01, ***p < 0.001, and ****p < 0.0001).

Investigations conducted on h-PBMCs to evaluate the anti-inflammatory potential of WLO and AAs demonstrated that these substances did not have any effects on pro-inflammatory cytokines. The safety of WLO and AAs was confirmed by a viability assay that demonstrated that neither WLO nor AAs reduced viable cell counts (Supplementary Figure 1). According to the results shown in Figure 3, under basal conditions the secretion of IL-6 was significantly reduced following exposure to both WLO and AAs while TNF-α release decreased only in the presence of 20 or 40 μg/mL of WLO and IFN-γ release was reduced only at 20 μg/mL of WLO. When tested in a model of LPS-induced inflammation, IL-6 and IFN-γ release was reduced significantly by WLO, while TNF-α release was unaffected. Under LPS-stimulated conditions, AAs increased the release of IL-6 at 0.015 and at 0.030 but not at 0.060 μg/mL. These last results overlap with those described by Qabaha et al. (2016) who reported that *Eucalyptus* spp. and *P*. *lentiscus* leaf extracts were able to reduce the secretion of IL-6 and TNF-α in a similar human cell model. These authors attributed the anti-inflammatory potential to gallic acid and to other minor phenolic compounds of LO. According to these authors, AAs were not confirmed to actively contribute to the observed anti-inflammatory ability of WLO since AAs increased the secretion of IL-6, but in a non-dose-dependent manner, while TNF-α and IFN-γ did not seem to be influenced by AAs.

## Discussion

It is estimated that 25,000 people die every year in Europe due to antibiotic-resistant bacterial infections. The United States Centers for Disease Control and Prevention conservatively estimate that more than 20,000 deaths a year in the United States are caused by antibiotic resistance. These numbers, although perhaps imprecise because resistant infections are more common in individuals on long courses of antibiotic treatment and it is difficult to ascertain whether resistance is the cause of death or if there is a simple correlation with a long period of antibiotic treatment and/or underlying sickness, are in any case useful for understanding the scale of the problem (Laxminarayan et al., 2013). The causes of antibiotic resistance are certainly complex and involve a human role at many levels (i.e., medical prescriptions, agricultural methods, use in livestock farms, and so on) but the consequences negatively affect the global population. Apart from new antibiotics, we need to better develop new treatment strategies, i.e., methods to stop plasmid replication (DeNap and Hergenrother, 2005) or resistance mechanisms such as efflux pump inhibitors (Kurinčič et al., 2012), and, despite the difficulties, the use of bacteriophages (Potera, 2013), along with constructing a better understanding of the possibilities offered by bacterial therapy, a strategy exploiting bacterial competition (Tagg and Dierksen, 2003). Among the different strategies, of note is the possible discovery of new antimicrobials found as botanical secondary metabolites. Phenols, tannins, terpenoids, alkaloids, flavonoids, and others may in fact possess specific biological activities against many pathogenic microorganisms. An example of this is the antibiotic role played by the alkaloid berberine against *Vibrio cholerae* and *Escherichia coli* (Khin-Maung-U et al., 1985). Another example are the studies performed on the non-edible component, essential oil from mastic branches and leaves, of lentisk (*P*. *lentiscus*) demonstrating a certain antibacterial activity (Piras et al., 2017). In addition, from this last botanical, but using the edible component like the cold-pressed berry oil (LO), a specific and differentiated anti-streptococcal activity has recently been observed (Orrù et al., 2017). In detail, *S*. *mitis* and *S*. *intermedius* were sensitive to LO, while *S*. *pyogenes*, *S*. *agalactiae*, and *S*. *mutans* were only partially sensitive, and *S*. *salivarius* was insensitive. According to the authors, LO contains at least two groups of antibacterial substances. The first group of substances, which is probably highly active in *S*. *intermedius* and *S*. *mitis*, is likely represented by phenols and some UFAs. A second group represented by fatty acid esters (FAEs) lacking a direct antibacterial activity exerts an antimicrobial effect on *S*. *pyogenes*, *S*. *agalactiae*, and *S*. *mutans* after being metabolized by bacterial esterase to UFAs. Conversely, the presence of a specific hydratase in *S*. *salivarius* metabolizes these UFAs to ineffective saturated fatty acids, keeping the bacteria alive.

As *P*. *lentiscus* belongs to the family Anacardiaceae, reported to contain antimicrobial compounds known as AAs (Salehi et al., 2019), we postulated that this fraction of substances could also be involved in the antimicrobial effect observed. We hypothesized that an AA-enriched fraction of LO would have a better antibiotic profile than LO. Winterization is a simple and cheap way of removing a wide array of fatty substances from vegetable oils. By applying the procedures described to winterize vegetable oils (Nergiz and Celikkale, 2011; Watari et al., 2020), we obtained a product enriched in AAs that we have used here to assess its possible antimicrobial effects and to further obtain a pure mixture of AAs. We focused our tests on those streptococcal pathogens (*S*. *pyogenes* and *S*. *pneumoniae*) involved in sore throat and middle ear pathologies and on two other non-streptococcal species often provoking pathology in the middle ear (*M*. *catarrhalis* and *H*. *influenzae*). We decided to investigate within this field due to the reported complete insensitivity of *S*. *salivarius* K12 and *S*. *salivarius* M18 to LO. In fact, both *S*. *salivarius* strains have been clinically reported to compete with oral pathogens, reducing the recurrency of pharyngitis, tonsillitis, middle ear pathologies, and tooth decay both in children and in adults (Di Pierro et al., 2013, 2015, 2016, 2018). We were then very interested in investigating the possibility that WLO could be effective against oral pathogens while being ineffective or less effective on oral commensals. Considering WLO as a possible adjuvant to antibiotics, we observed that both *M*. *catarrhalis* and *S*. *pneumoniae* were completely killed by using half the concentration of AC needed if WLO was added at 800 μg/mL (see Table 3). Moreover, we suppose that the antimicrobial role observed for WLO could also be explained by considering its AA content. Even if their mixture, in WLO, corresponded to only 0.0064%, they indeed exerted a similar effect to WLO on our strains with the MIC values for *M*. *catarrhalis*, *S*. *pneumoniae*, and *S*. *pyogenes*, respectively, lower than those observed for *S*. *salivarius* K12 by 84, 75, and 50% (Table 2).

The distinction between bactericidal and bacteriostatic antibiotics is a successful concept to discriminate antibiotics that kill bacteria from antibiotics that inhibit bacterial growth. This classification, bactericidal vs. bacteriostatic, is generally applied and recognized. The intuitively understandable concept between the two groups suggests that bactericidal drugs have more powerful antibacterial action and can kill bacteria. In contrast, bacteriostatic drugs are assumed to require the action of the immune system to clear bacteria and are therefore thought to be less effective without an efficient phagocytic response. This theoretical model has led to the recommendation that severely ill and immunosuppressed patients with bacterial infections should be treated with bactericidal antibiotics. Anyway, even if there are no clinical data supporting the concept of bacteriostatic vs. bactericidal antibiotics, likely due to the difficulties of assessing a drug class effect in a clinically meaningful way, in our tests a direct comparison between the MIC and the MBC values (Table 2 vs. Table 4) suggests us to consider both WLO and AAs as possible bactericidal agents, at least as regards to the pathogenic strains. Differently, WLO and AAs demonstrated a more likely bacteriostatic effect on probiotics. We have given up to the idea of evaluating our actives after washing out them from the tests because of their lipophilic nature that should have affected negatively our methodology with the risk of providing false results.

The results presented in Table 3 once again support the idea that AAs are one of the possible antimicrobial agents present in WLO. In fact, the coactive effect with antibiotics highlighted for WLO seemed to be even more effective in the presence of AAs. The advantage of purifying AAs is mainly linked to the final concentration needed to inhibit pathogen growth. In any case, notwithstanding the better antibiotic effect seen with AAs vs. WLO, the two probiotic strains *S*. *salivarius* K12 and M18 survived both at higher percentages of antibiotics used as a single treatment and when tested in combination with WLO or AAs, compared to *S*. *pyogenes* LMG 21599, *S*. *pneumoniae* DSM 20566, and *M*. *catarrhalis* DSM 9143. Moreover, *H*. *influenzae* DSM 4690 required a half-dose of antibiotics when combined with 256 μg/mL AAs, and even if the MIC values are higher than for the other microorganisms considered, these results suggest that AAs should be evaluated within the context of clinical approaches to infective diseases as co-adjuvants with antibiotics.

It is the case that our results do not allow us to confirm whether the apparently selective antimicrobial role of WLO is due to a direct toxic action on pathogens or if this is due to an effect exerted over their microbial biofilms. In a very preliminary way, we have thus investigated, only with *S*. *salivarius* probiotic strains and with *S*. *pyogenes*, the effects of WLO (at 0.39, 0.78, 1.56, and 3.12%) using Congo red agar prepared with BHI (52 g/L), saccharose (36 g/L), and Congo red (0.08%). Visual assessment of agar cultures showed greater biofilm formation in probiotics cultures than in *S*. *pyogenes* (Supplementary Figure 2). In proportion to the dosage of WLO added to the cultures, and especially at the highest concentration of WLO (3.12%), by evaluating the color variation from dark red to reddish, we seem to have observed, along with a reduction in the number of colonies, a reduction in the capability of bacteria to produce biofilm, both in probiotics and in *S*. *pyogenes* (Supplementary Figures 3–5). If this were to be confirmed using other types of tests, including quorum sensing and electron microscopy tests, a possible anti-biofilm effect of WLO could be taken into consideration; this would suggest that the synergy observed between WLO and antibiotics could be explained by considering WLO as not (only) a direct antimicrobial agent but also as a tool able to damage the bacterial biofilm. Since determination of the potential mechanism(s) of action of WLO was not the primary aim of our investigation, these preliminary results only act to suggest a possible role for WLO against bacterial biofilm. Further assessment in this regard is therefore required to confirm any proposed mechanism of action.

Apart from this, our data suggest potential benefits from the use of WLO, alone or together with antibiotics, on pharyngeal and/or nasal mucosae. Therefore, we considered it important to assess the role played by such an active in inflammation. Our results demonstrated a possible effect of WLO in restraining the release of inflammatory cytokines such as IL-6 and IFN-γ when h-PBMCs were challenged with LPS. In terms of anti-inflammatory properties, our results demonstrated WLO to be a more promising agent compared to AAs.

In conclusion, despite the biases due to the use of an *in vitro* model and the limited number of pathogenic strains used in our tests, our preliminary results indicate that WLO: (1) differently to LO, demonstrates an antimicrobial role for *S*. *pyogenes* also, while still showing poor effectiveness with respect to *S*. *salivarius*; (2) shows a clear antimicrobial role also with respect to *S*. *pneumoniae* and *M*. *catarrhalis*; (3) when tested along with antibiotics, especially when tested with AC, they seem to act as an adjuvant antimicrobial; (4) exerts its antimicrobial role also because of its AA content; (5) could be co-administered along with strains of *S*. *salivarius*, at least with strains such as K12 and M18, in the attempt to prevent and/or counteract infections caused by common pharyngeal and middle ear pathogens such as *S*. *pyogenes*, *S*. *pneumoniae*, *M*. *catarrhalis*, and *H*. *influenzae*; (6) if used locally, should not considerably reduce the presence of endogenous strains of *S*. *salivarius* in the oral microbiota; and (7) seems to reduce, at least in our test conditions, the release of inflammatory cytokines.

## Data Availability Statement

The raw data supporting the conclusions of this article will be made available by the authors, without undue reservation.

## Author Contributions

FD, VS, ME, and AB conceived the idea, wrote, revised, and edited the manuscript. SeG, MF, and VS performed the microbiological tests on LO and derivatives. SiG performed the chemical test on LO. All authors read and made final approval of the manuscript.

## Conflict of Interest

FD and AB are scientific consultants of Pharmextracta. VS, SeG, MF, and ME work for AAT, a laboratory having a scientific collaboration with Pharmextracta. The remaining author declare that the research was conducted in the absence of any commercial or financial relationships that could be construed as a potential conflict of interest.

## Supplementary Material

Supplementary Figure 1 Trypan blue assay for viable cell determination.

Supplementary Figure 2 *S*. *salivarius* strains K12 (A) and M18 (B) and *S*. *pyogenes* (C) in Congo red agar.

Supplementary Figure 3 *S*. *pyogenes* in Congo red agar with basal medium **(A)** and with WLO at 0.39% **(B)**, 0.78% **(C)**, 1.56% **(D)**, and 3.12% **(E)**.

Supplementary Figure 4 *S*. *salivarius* M18 in Congo red agar with basal medium **(A)** and with WLO at 0.39% **(B)**, 0.78% **(C)**, 1.56% **(D)**, and 3.12%.

Supplementary Figure 5 *S*. *salivarius* K12 in Congo red agar with basal medium **(A)** and with WLO at 0.39% **(B)**, 0.78% **(C)**, 1.56% **(D)**, and 3.12%.
