# Supplementary figures and images for: Antibacterial Activity of a Fractionated Pistacia lentiscus Oil Against Pharyngeal and Ear Pathogens, Alone or in Combination With Antibiotics
Source: Front Microbiol. 2021 Jun 17;12:686942. doi: 10.3389/fmicb.2021.686942 (PMC8247648; doi:10.3389/fmicb.2021.686942)

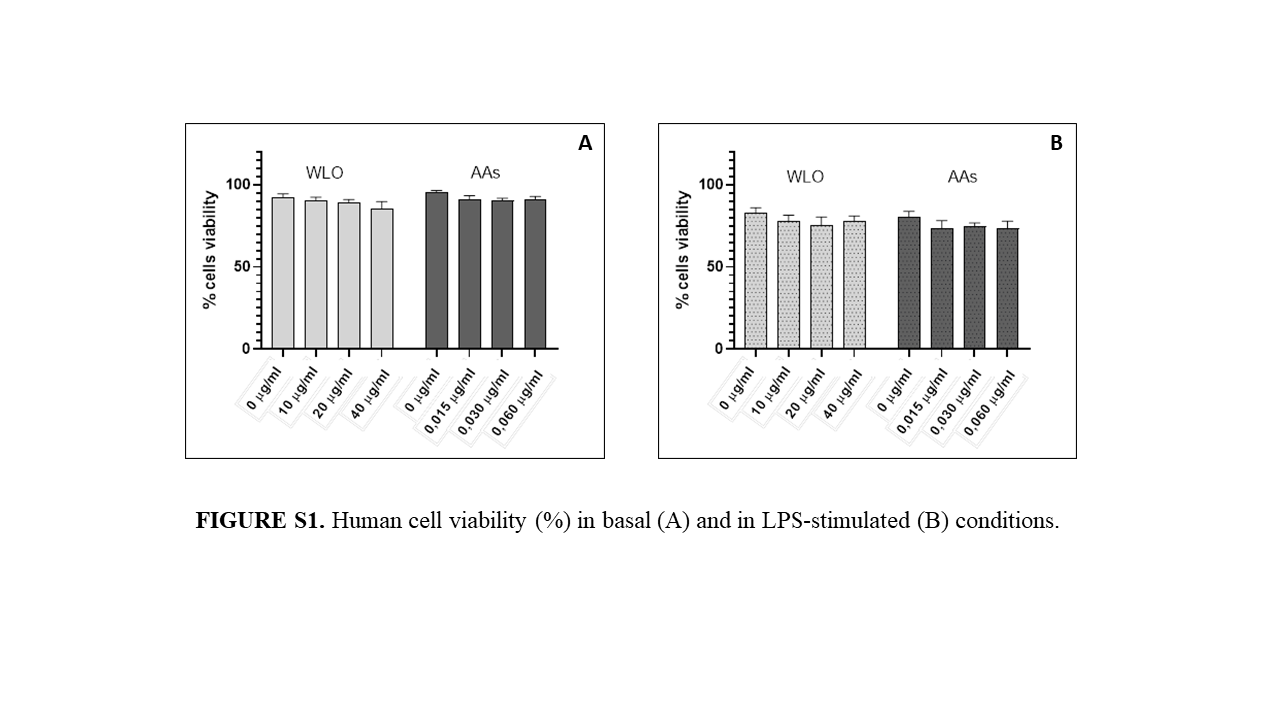

Supplement: Supplementary Figure 1 — Trypan blue assay for viable cell determination. [file Image_1.TIF]

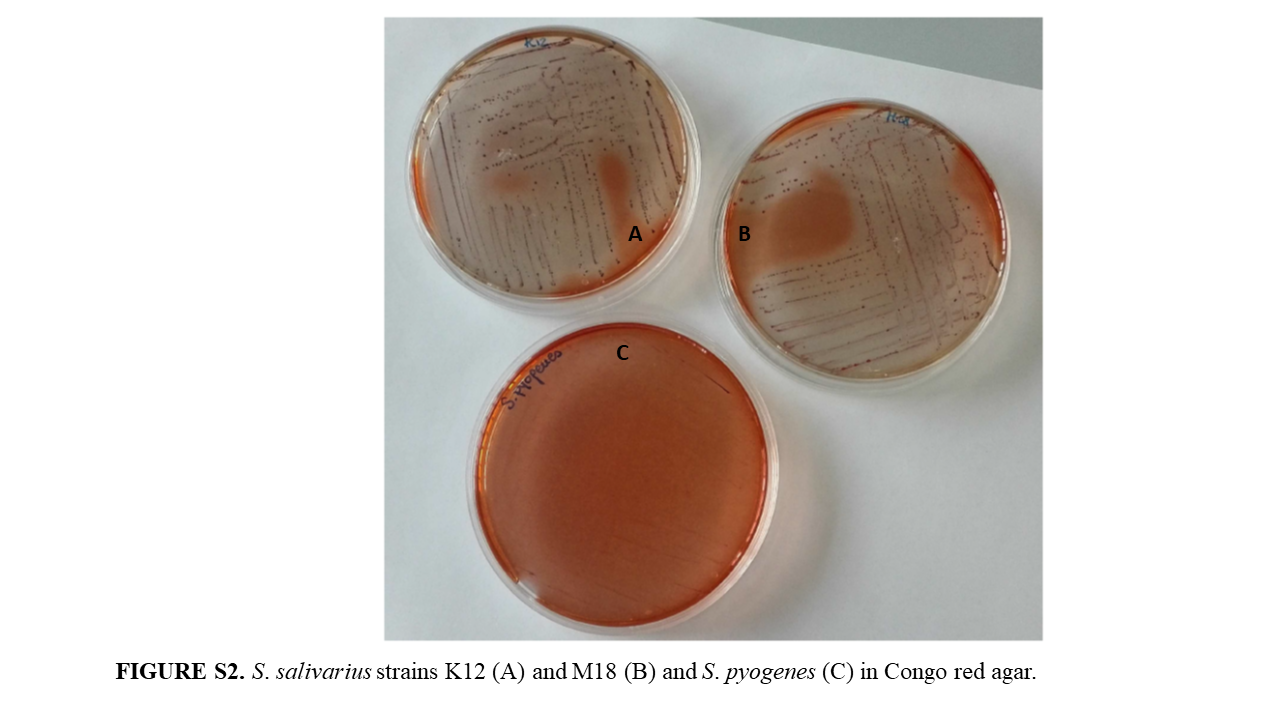

Supplement: Supplementary Figure 2 — S. salivarius strains K12 (A) and M18 (B) and S. pyogenes (C) in Congo red agar. [file Image_2.TIF]

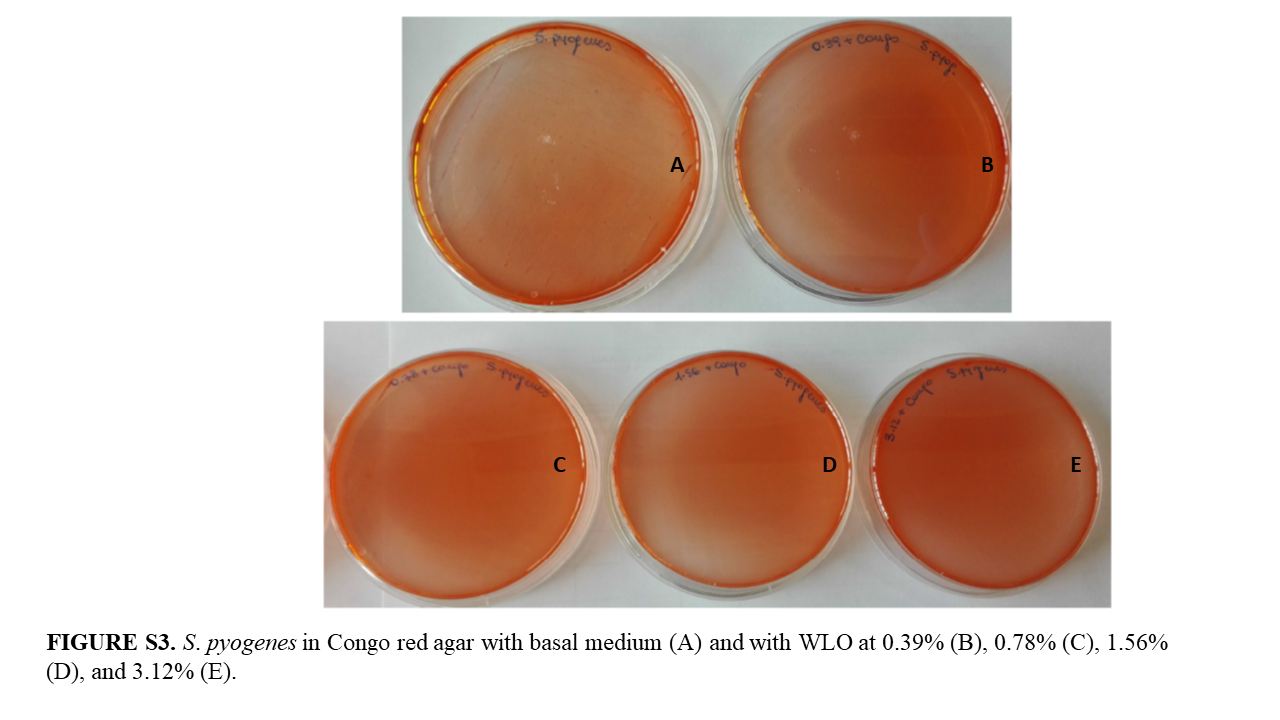

Supplement: Supplementary Figure 3 — S. pyogenes in Congo red agar with basal medium (A) and with WLO at 0.39% (B), 0.78% (C), 1.56% (D), and 3.12% (E). [file Image_3.TIF]

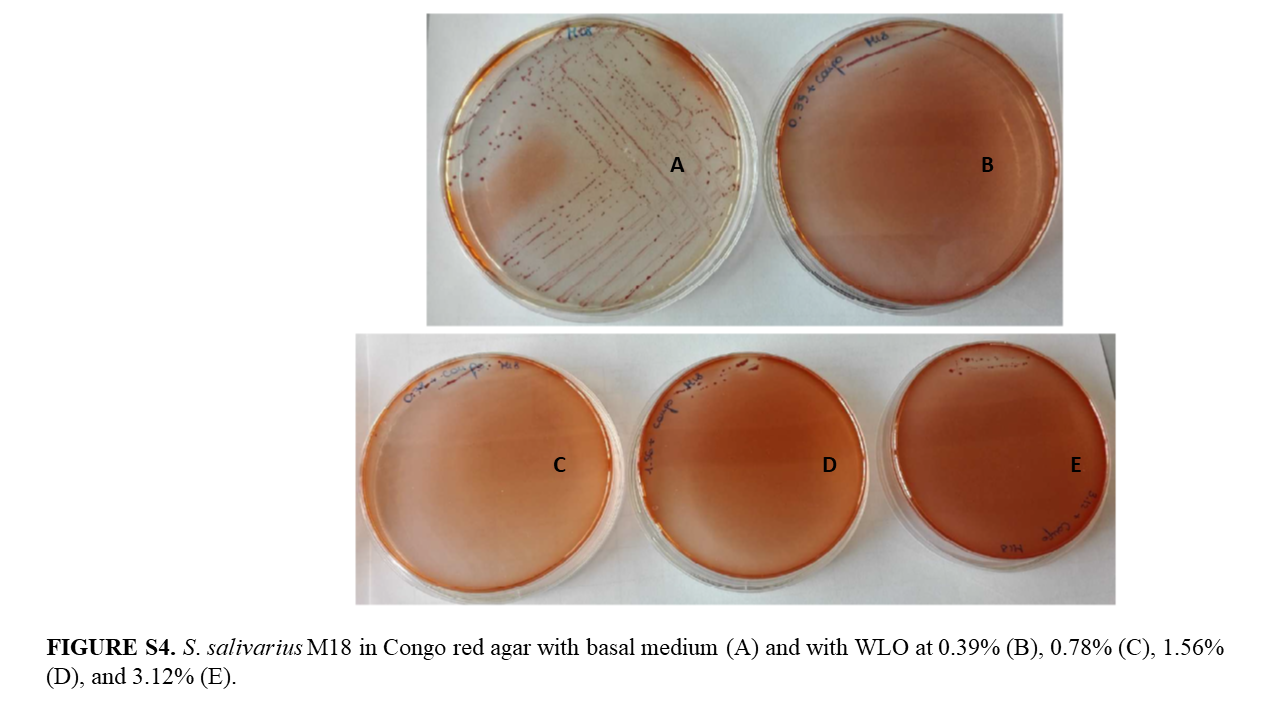

Supplement: Supplementary Figure 4 — S. salivarius M18 in Congo red agar with basal medium (A) and with WLO at 0.39% (B), 0.78% (C), 1.56% (D), and 3.12%. [file Image_4.TIF]

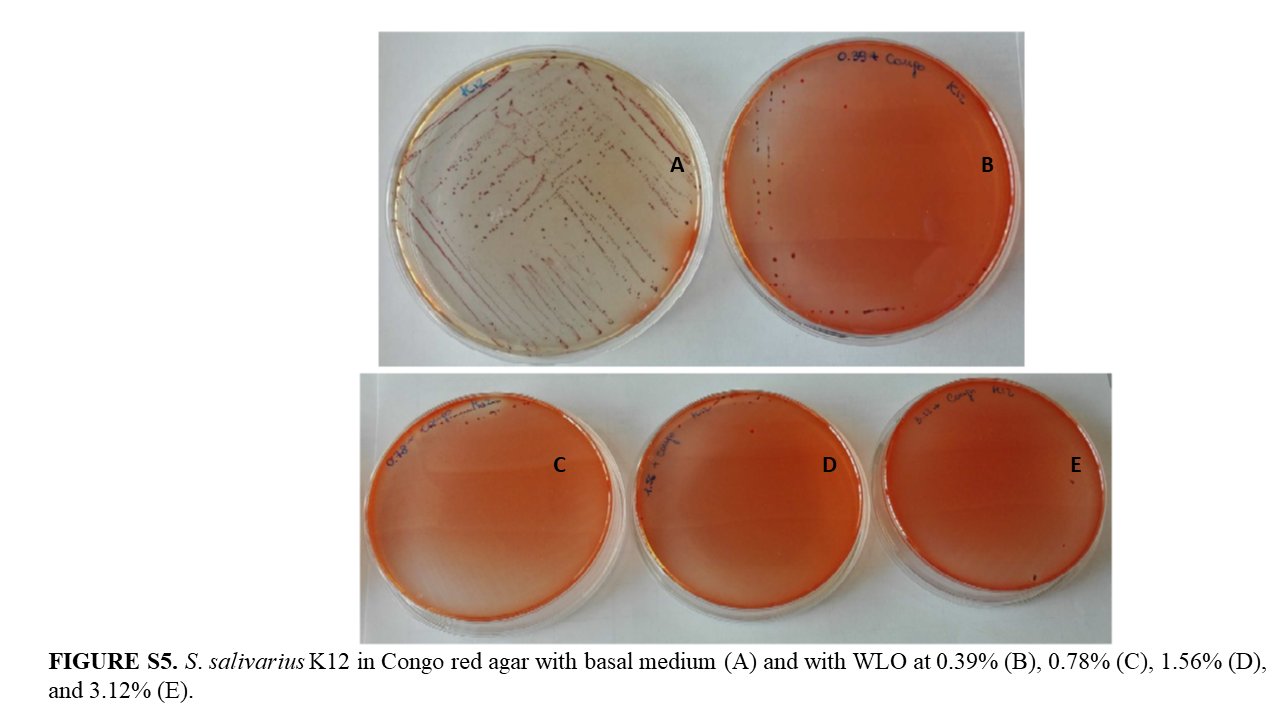

Supplement: Supplementary Figure 5 — S. salivarius K12 in Congo red agar with basal medium (A) and with WLO at 0.39% (B), 0.78% (C), 1.56% (D), and 3.12%. [file Image_5.TIF]
